# Characterization of Natural Products as Inhibitors of Shikimate Dehydrogenase from Methicillin-Resistant *Staphylococcus aureus*: Kinetic and Molecular Dynamics Simulations, and Biological Activity Studies

**DOI:** 10.3390/biom15081137

**Published:** 2025-08-06

**Authors:** Noé Fabián Corral-Rodríguez, Valeria Itzel Moreno-Contreras, Erick Sierra-Campos, Mónica Valdez-Solana, Jorge Cisneros-Martínez, Alfredo Téllez-Valencia, Claudia Avitia-Domínguez

**Affiliations:** 1 Facultad de Medicina y Nutrición, Universidad Juárez del Estado de Durango, Av. Universidad y Fanny Anitua S/N, Durango 34000, Mexico; noe.corral.96@outlook.com (N.F.C.-R.); p363963@uach.mx (V.I.M.-C.); jorgecisner10@yahoo.com.mx (J.C.-M.); 2 Facultad de Ciencias Químicas, Universidad Juárez del Estado de Durango Campus Gómez Palacio, Avenida Artículo 123 S/N, Fracc. Filadelfia, Gómez Palacio 35010, Mexico; ericksier@ujed.mx (E.S.-C.); valdezandyval@gmail.com (M.V.-S.)

**Keywords:** drug discovery, natural products, methicillin-resistant *S. aureus*, shikimate pathway, shikimate dehydrogenase

## Abstract

Antibiotic resistance is considered to be one of the most complex health obstacles of our time. Methicillin-resistant *Staphylococcus aureus* (MRSA) represents a global health challenge due to its broad treatment resistance capacity, resulting in high mortality rates. The shikimate pathway (SP) is responsible for the biosynthesis of chorismate from glycolysis and pentose phosphate pathway intermediates. This pathway plays a crucial role in producing aromatic amino acids, folates, ubiquinone, and other secondary metabolites in bacteria. Notably, SP is absent in humans, which makes it a specific and potential therapeutic target to explore for discovering new antibiotics against MRSA. The present study characterized in vitro and in silico natural products as inhibitors of the shikimate dehydrogenase from methicillin-resistant *S. aureus* (*Sa*SDH). The results showed that, from the set of compounds studied, phloridzin, rutin, and caffeic acid were the most potent inhibitors of *Sa*SDH, with IC_50_ values of 140, 160, and 240 µM, respectively. Furthermore, phloridzin showed a mixed-type inhibition mechanism, whilst rutin and caffeic acid showed non-competitive mechanisms. The structural characterization of the *Sa*SDH–inhibitor complex indicated that these compounds interacted with amino acids from the catalytic site and formed stable complexes. In biological activity studies against MRSA, caffeic acid showed an MIC of 2.2 mg/mL. Taken together, these data encourage using these compounds as a starting point for developing new antibiotics based on natural products against MRSA.

## 1. Introduction

Antibiotic resistance is considered to be one of the most complex health issues of our time [1]. It is estimated that, over the coming years, the efficiency of medical treatments will decrease, causing prolonged infections, an increase in medical expenses, and a substantial increase in mortality rates [1]. On the other hand, the overexposure of current antibiotics to non-meritorious situations increases the risk of the evolution of new emerging bacterial infections [2]. A key example of this scenario occurred during the COVID-19 pandemic, where a sudden increase in antibiotic consumption was believed to have occurred due to the limited availability of specific medical strategies [3].

Despite exploring potential antibiotic treatments constituting a fundamental aspect of modern medical research, some bacterial agents represent a challenge for public health [4,5]. Due to its wide landscape of pathogenicity reflected in abscesses, sepsis, pneumonia, bacteremia, endocarditis, osteomyelitis, and other issues affecting skin, soft tissue, bone, joint, bloodstream, systemic, device-related, and implant-related infections, methicillin-resistant *Staphylococcus aureus* (MRSA) represents a global health challenge due to its broad resistance to a vast number of current treatments, resulting in high mortality rates [4,6]. In this context, the search for alternatives for developing new drugs has focused on exploring multiple medical targets, with the Shikimate pathway being a strategic entry route [7,8,9].

The importance of the Shikimate pathway (SP) stems from the biosynthesis of chorismate from phosphoenolpyruvate (PEP) condensation derived from glycolysis and erythrose 4-phosphate in turn derived from the pentose phosphate pathway [8]. This pathway is present in fungi, plants, some protozoa, algae, apicomplexan parasites, and bacteria but absent in humans. Therefore, it represents a potential therapeutic target for developing antibiotics [10].

Chorismate synthesis via the SP consists of seven key enzymatic reactions used for the biochemical formation of this essential precursor through synthesizing amino acids, folates, ubiquinone, and other secondary molecules [7]. One of the enzymatic reactions of the SP is carried out by the shikimate 5-dehydrogenase (EC 1.1.1.25) (SDH) (responsible for the fourth reaction of the pathway), which is the reversible reduction of 3-dehydroshikimate to shikimate using NADPH as a cofactor [11].

Inhibiting SDH represents an opportunity to interrupt the SP, which could represent a potential specific therapeutic target for infections caused by MRSA [12]. Recently, inhibitors of this enzyme have been reported, including some natural compounds [13,14,15]. Some phenolic compounds from plants and fruits have shown antioxidant, anti-inflammatory, and anticancer capacities and antibiotic properties [16,17,18]. Unlike many synthetic drugs, natural compounds present a lower risk of side effects, making them attractive options for developing innovative antimicrobial therapies [19]. On the other hand, their natural origin may enhance compatibility with biological organisms; in addition, their chemical diversity may provide specificity, potentially reducing antibacterial resistance [20,21,22].

Recent in vitro and in silico approaches have highlighted significant potential for characterizing and validating potential SDH inhibitors in pathogens such as *Mycobacterium tuberculosis* [23,24] and *Escherichia coli* [13], as well as MRSA [25,26]. These models, in combination, present a more efficient and detailed exploration of compounds with clinical potential, which may hasten the discovery of potential antibiotics, along with reduced research costs [27,28].

Additionally, computational tools have become a key, cost-effective approach for exploring biomolecular interactions and guiding drug discovery. In silico tools, such as molecular docking and dynamics simulations, offer rapid insights into how therapeutic targets interact with and conform to potential ligands, thereby accelerating early-stage drug discovery. Molecular docking, in general, a static prediction of binding modes, facilitates receptor-ligand binding prediction in virtual screening of large chemical libraries, also would be useful for lead optimization, and structure-based drug design [29,30]. In parallel, molecular dynamics simulations, a technique that permits to predict how atomic structures behave and respond over time, has several applications such as to study protein flexibility or protein-ligand complex stability, providing valuable insights into ligand-target interactions, which is a valuable information in the drug design process [31]. In combination, molecular docking and dynamics provide a deeper understanding of binding interactions and stability, reinforcing rational, structure-based drug discovery methods [29,30].

In this regard, the present study characterizes in vitro and in silico natural products as inhibitors of the SDH from methicillin-resistant *S. aureus* (*Sa*SDH). The molecules reported here present binding to the active site of the enzyme, and interactions with relevant residues for catalysis are shown, as well as the inhibition activity of the compounds and the suppression of MRSA growth, considered as bioactive molecules with antimicrobial potential.

## 2. Materials and Methods

### 2.1. Purification of the Recombinant SaSDH

The purification of *Sa*SDH was performed following the method employed by Avitia and colleagues [26]. In brief, an *E. coli* BL21(DE3) strain was transformed with the plasmid that contained the *AroE* gene and grown in 250 mL of Luria–Bertani medium supplemented with kanamycin (50 µg/mL). Overexpression was performed by inducing the culture when it reached an OD_600_ nm of 0.5 Absorbance Units (AU). Henceforth, induction was carried out with 0.4 mM of IPTG and incubation for 3 h at 37 °C, with shaking at 200 rpm. Subsequently, the cells were harvested and resuspended in buffer A (50 mM Tris-HCl, pH 8.0, 0.5 M NaCl, 5% (*v*/*v*) glycerol) supplemented with 200 µM of phenylmethylsulfonyl fluoride and then sonicated. The cellular debris was removed via centrifugation at 25,000 rpm at 4 °C for 30 min. The *Sa*SDH supernatant was loaded onto a Ni-Agarose affinity column previously equilibrated with buffer A. *Sa*SDH was eluted with buffer A supplemented with concentrations of imidazole increasing from 20 to 300 mM. The protein concentration was determined via the Bradford method [32].

### 2.2. Enzymatic Activity Assays

The enzymatic activity of *Sa*SDH was analyzed by reducing NADP^+^ to NADPH at 340 nm and 25 °C. Reaction mixtures containing 100 mM of Tris-HCl, pH 8.0, 250 µM of shikimate, and 250 µM of NADP^+^ were made. The reaction was started by adding *Sa*SDH to a 0.05 mg/mL concentration. The enzyme activity was determined spectrophotometrically using the molar extinction coefficient of NADP^+^ (ε = 6.2 × 10^−3^ M^−1^ cm^−1^).

### 2.3. Inhibition Screening Assays

The inhibition percentage was determined by measuring enzyme activity under the conditions previously described by adding each compound from an *in-house* natural product derivative chemical library (40 compounds) to the reaction mixture at quantities of 500 μM; these compounds had been previously dissolved in Dimethyl Sulfoxide (DMSO), keeping 10% DMSO as final concentration. The percentage inhibition was calculated by adjusting the data from the following equation [33]:

$$Inhibition\;\left(\%\right)=\frac{\left(A0-A1\right)}{A0}\;\times 100$$

where the following definitions are used:

*A*0: Absorbance of the negative control;

*A*1: Absorbance of the test sample.

### 2.4. Evaluation of IC_50_ Values

The concentration that inhibited enzyme activity by 50% (IC_50_) was determined using curves at different concentrations of the selected inhibitor and saturating concentrations of each substrate. The IC_50_ was determined by adjusting the data to the dose–response equation [34].

### 2.5. Inhibition Mechanism Characterization

Time–course curves were created using enzyme kinetics to determine the inhibition mechanism of the molecules with the highest inhibitory capacity. These assays were performed by measuring enzyme activity at the saturating concentrations of the shikimate substrates and NADP^+^ (250 µM for both) and in the presence of different concentrations of the inhibitor (70 µM to 250 µM), following the reaction for 1200 s at an absorbance of 340 nm and a temperature of 25 °C. The assays were performed in duplicate. The data obtained were analyzed using GraphPad Prism v8 and modified to the Lambert W function [35,36].

$${\left[P\right]}_{t}={\left[S\right]}_{0}-\ln\left(\frac{x}{\ln\left(2.4\times \frac{x}{\ln\left(1+2.4*x\right)}\right)}\right)-0.45869\times ln\left(2*\frac{x}{ln\left(1+2*x\right)}\right)$$

where

$$x=\frac{\left[S\right]O}{Km}\times exp\left(\frac{\left[S\right]O-Vm*t}{Km}\right)$$

### 2.6. Evaluation of Biological Activity

The Minimum Inhibitory Concentration (MIC) of the most potent *Sa*SDH inhibitors was determined via biological activity assays on a methicillin-resistant *Staphylococcus aureus* strain (ATCC^®^ BAA-1720TM, Manassas, VA, USA) using the plate microdilution method [37]. Test compounds were dissolved in 2% DMSO. A Muller–Hinton agar plate was inoculated with the MRSA strain and incubated at 37 °C for 24 h. A preculture was then prepared in Muller–Hinton broth adjusted to 0.5 McFarland (0.08–0.1 AU) and diluted to 1:100. Subsequently, the ELISA plate wells were inoculated with this dilution and the test compound at various concentrations, reaching a final volume of 200 µL in each well. Therefore, the plate was incubated at 37 °C and 200 rpm for 16–20 h. The optical density at 600 nm was measured, and the inhibition percentage was calculated by using the untreated control as 100% growth. Finally, the MIC was defined as the lowest compound concentration preventing visible bacterial growth. The assays were performed by triplicate.

### 2.7. Molecular Docking

A molecular docking analysis of the enzyme–inhibitor interaction was conducted with Autodock Vina v.1.2.5 [38] using a gradient-based local search genetic algorithm to predict the binding mode of the inhibitors, with random ligand conformations, a spacing of 1, an exhaustiveness of 100, and considering a rigid receptor model. The selected compounds were prepared by adding hydrogen atoms and assigning partial charges through the Gasteiger–Marsili algorithm [39]. A homology-generated model reported by our research group was utilized for molecular docking due to the lack of a crystallographic structure of *Sa*SDH [26]. Preparing the *Sa*SDH protein involved adding hydrogen atoms and assigning partial charges, and the box was constructed by selecting the residues involved in the shikimate binding site, Val5, Ser13, Ser15, Asn58, Ile59, Thr60, Lys64, Asn85, Asp100, Phe236, and Gln239, as well as the residues related to the NADP^+^ binding site, Lys64, Glu65, Ala83, Ala125, Gly126, Gly127, Ala128, Ser129, Lys130, Ile132, Asn148, Arg149, Arg153, Leu166, Thr182, Thr183, Pro184, Met187, Ile194, Ile209, and Met235. Finally, the center coordinates were assigned (x = 51.04, y = 36.64, z = 42.84), and the box size was 24 × 28 × 34 Å^3^. Post-docking interaction analyses were accessed using the PLIP (Protein-Ligand Interaction Profiler) server [40,41].

### 2.8. Molecular Dynamics Simulations

The ligands were parametrized using the ACPYPE server [42] with an AMBER force field [43]. All simulations were performed using a AMBER99 force field [44]. Initially, energy minimization was carried out using 1000 steepest-descent cycles. Initial velocities were assigned from Maxwell distribution at 10 K, followed by a gradual temperature increase to 300 K. Subsequently, we performed 50,000 steps of molecular dynamics to carry out canonical (NVT) [45] and isothermical–isobaric (NPT) [46] simulations (with isotropic position scaling at 300 K and 1 atm pressure, while the simulation system was solvated in a cubic box (100 Å) filled with the simple point charge water model [47] (ranging from 16,763 to 20,600 water molecules) and neutralized with 0.15 M of Na^+^ and Cl^−^ ions (57 Na^+^ and 50 Cl^−^ for SaSDH-inhibitor complex; 68 Na^+^ and 61 Cl^−^ for SaSDH alone). Finally, the complete molecular dynamic simulations of 100 ns at 300 K without restrictions and a timestep of 2 fs, Particle Mesh Ewald (PME) for long-range electrostatics, velocity rescale temperature coupling with a time constant (τ) of 0.1 ps, periodic boundary conditions, the Parrinello–Rahman method for pressure coupling with a τ of 2 ps, and the Verlet cutoff scheme for non-bonded interactions, were carried out using GROMACS 2022 (Groningen Biomolecular Sciences and Biotechnology Institute, Groningen, The Netherlands) [48], obtaining 10,000 conformations that were saved every 5000 steps. Further analyses were carried out by calculating the root mean square deviation (RMSD), the root mean square fluctuation (RMSF), and the radius of gyration (Rg).

### 2.9. Linear Interaction Energy Calculation

Binding free energies of each complex were obtained from the last 50 ns of simulation time according to the linear interaction energy (LIE) method, that relies on a linear approximation of the interaction energy between the ligand and the protein, using a combination of electrostatic and van der Waals interactions. Furthermore, it provides reasonably accurate binding free energy predictions and is computationally less expensive than other free energy calculation methods like MMPBSA [49]. The value was calculated using the following equation [50,51,52]:

∆Gbind = α[(VLJ)bound − (VLJ)free] + β[(VCL)bound − (VCL)free] + γ

where (VLJ)bound represents the average Lennard-Jones energy representing ligand–protein interaction, (VLJ)free represents the average Lennard-Jones energy for ligand–solvent interaction, (VCL)bound represents the average electrostatic energy for ligand–protein interaction, (VCL)free represents the average electrostatic energy for ligand–solvent interaction, and the LIE coefficients are represented by α, β, and γ, which for small drug-like ligands are equivalent to α = 0.18, β = 0.50, and γ = 0.00.

### 2.10. Drug-like and ADME-Tox Properties

The physicochemical and toxicological properties of the inhibitors were assessed using the online servers PreADMET [53] and SWISSADME [54] and the software DataWarrior v.4.07.02 [55]. Drug-like properties were predicted based on Lipinski’s rules of five (Ro5) and drug-like rules. Additionally, ADME-Tox properties were predicted, such as absorption in the human intestine, binding to plasma proteins, and penetration of the blood–brain barrier. Moreover, toxicological predictions were conducted to assess potential mutagenicity, carcinogenicity, irritant effects, reproduction effects, and G-glycoprotein and CYP450 inhibition.

## 3. Results

### 3.1. Natural Products Derivatives Screening

In the search for new SaSDH inhibitors, an *in-house* chemical library of 40 natural product derivatives was screened against SaSDH activity. Our findings indicate that among the 40 molecules evaluated, the compounds phloridzin (93% of inhibition), rutin (87% of inhibition), and caffeic acid (70% of inhibition) inhibited SaSDH using a 500 µM concentration. The remaining compounds showed percentages of inhibition between 30 and 45% (5 compounds), 20 and 29% (3 compounds), and 10–19% (16 compounds), while the rest (13 compounds) showed under 9% or no inhibition (Table S1). The chemical structures of the eight compounds with the highest inhibition activities are shown in Table 1.

### 3.2. SaSDH Inhibitor Characterization

**Figure 1:**
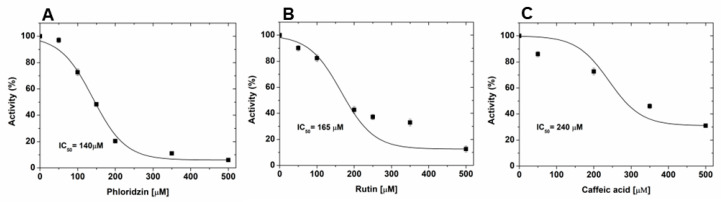
IC_50_ determination graphs: (**A**) phloridzin, (**B**) rutin, and (**C**) caffeic acid. The plots show the mean ± SD of the assays performed in triplicate.

Once the screening was completed, the three compounds with the highest inhibition capabilities were selected for further characterization. Firstly, the concentration that inhibited 50% of enzyme activity (IC_50_) was determined. The data showed that phloridzin was the most potent, followed by rutin and caffeic acid, obtaining IC_50_ values of 140, 165, and 240 µM, respectively (Figure 1). These values are in the range of other inhibitors reported for this enzyme [25,26].

Thereafter, their inhibition mechanism in SaSDH was determined through temporal courses. These assays measured enzyme activity at saturating concentrations of shikimate and NADP^+^ (250 µM each) with varying inhibitor concentrations (Figure S1). The data were adjusted to the Lambert W function for kinetic parameter (Vmax and Km) determination. As shown in Table 2, phloridzin decreased Vmax and increased Km compared to the control, indicating mixed inhibition with a predominantly competitive component. This suggests that the inhibitor binds to both the free enzyme and the enzyme–substrate complex with a higher affinity for the free enzyme.

Regarding rutin and caffeic acid, the Vmax decreased, but Km values remained constant. This behavior indicated that both inhibitors affect enzyme catalysis by reducing the number of functional enzyme molecules that can catalyze the reaction. Therefore, a non-competitive inhibition mechanism could be deduced, since both have the same affinity for the free enzyme as for the enzyme–substrate complex.

### 3.3. Minimum Inhibitory Concentration (MIC) in MRSA

Furthermore, an important issue in searching for hit compounds is their biological activity. In this context, the antimicrobial activities of these natural product derivatives were evaluated against MRSA. The Minimum Inhibitory Concentration (MIC) was determined in the MRSA strain ATCC^®^ BAA-1720™. The maximum solubility of each compound in 2% DMSO was determined beforehand, as higher DMSO concentrations exerted antibacterial effects (unpublished data). Phloridzin and rutin showed low solubility in the Mueller–Hinton broth due to precipitation. Therefore, their MICs could not be determined.

**Figure 2:**
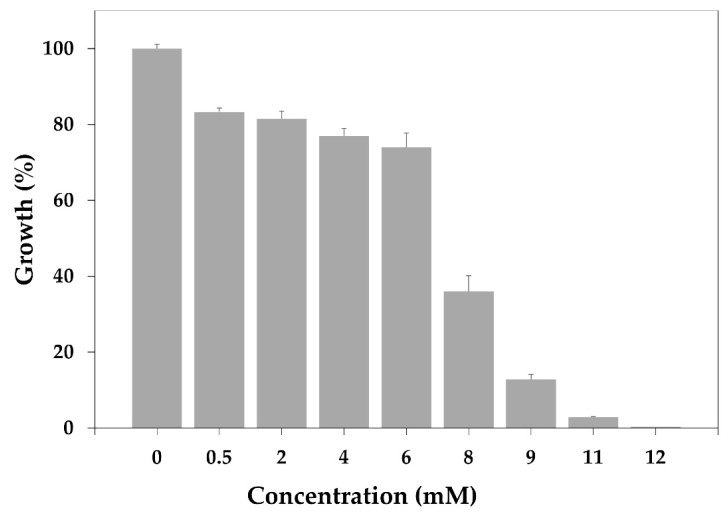
MRSA growth inhibition by different concentrations of caffeic acid. Bars represent the mean of triplicate measurements ± SD.

On the other hand, caffeic acid was soluble in the Mueller–Hinton broth at concentrations from 0.5 mM to 12 mM. The results showed that only at the highest concentrations tested was the growth of MRSA inhibited (Figure 2). From these data, a MIC of 12 mM was determined.

### 3.4. Molecular Docking Analysis

**Figure 3:**
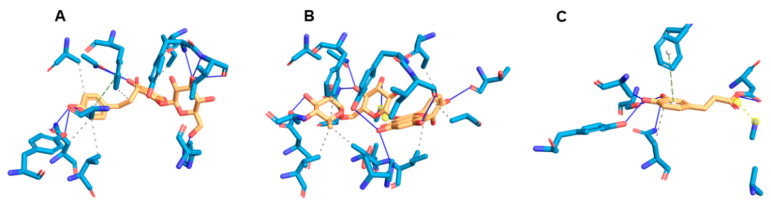
SaSDH–inhibitor interactions. (**A**) A 3D map of the SaSDH–phloridzin complex. (**B**) A 3D map of SaSDH–rutin. (**C**) A 3D map of SaSDH–caffeic acid complex. Hydrogen bond interactions are indicated with purple arrows. Polar residues are shown in sky blue, positively charged residues in navy blue, hydrophobic residues in green, and negatively charged residues in orange.

The study of enzyme–ligand complex interactions reveals useful information for future compound optimization, explaining how the inhibitor binds in the protein. For the SaSDH–phloridzin complex, data revealed a docking score of −8.9 kcal/mol, stabilized by three hydrogen bond interactions. One occurred between Ser13 and the ether group of the compound; another between Lys64, an essential residue involved in the catalysis, and the hydroxyl group at position five of the compound’s trihydroxy moiety; and the third between Tyr211 and the hydroxyl group on the linear side chain of the molecule (Figure 3A). The SaSDH–rutin complex presented a docking score of −10 kcal/mol, forming a hydrogen bond between Ser13 and the hydroxyl group at position 5 of the compound’s A-ring (Figure 3B). Finally, the SaSDH–caffeic acid complex obtained a docking score of −5.8 kcal/mol and was stabilized through three hydrogen bond interactions: one with the Leu14 residue and the hydroxyl group located in position 3, along with two interactions formed between Ser15 and Gln239 and the carboxyl group in the molecule (Figure 3C). A list of all interactions formed in each complex is shown in Table 3.

### 3.5. Molecular Dynamics Simulation Studies

#### 3.5.1. Root Mean Square Deviation, Root Mean Square Fluctuation, and Radius of Gyrate

**Figure 4:**
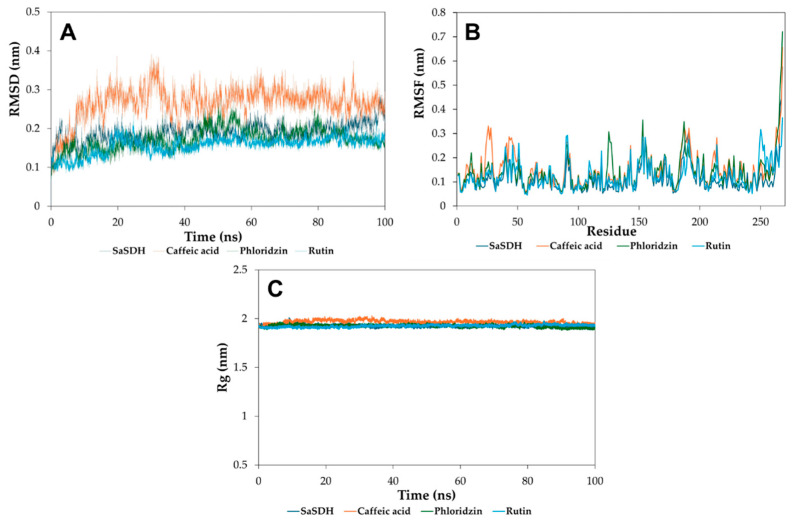
The RMSD (**A**), RMSF (**B**), and Rg (**C**) plots from the protein alone and in the complex with the three most potent inhibitors.

To gain more information related to the enzyme–inhibitor complex, molecular dynamics simulations (100 ns) were performed with phloridzin, rutin, and caffeic acid, as well as the protein alone. The results showed that the systems of protein alone, as well as the SaSDH–phloridzin and SaSDH–rutin complexes, reached stability after 20 ns, while only the SaSDH–caffeic acid complex was stable after 40 ns, as indicated by the root mean square deviation (RMSD) plots (Figure 4A). On the other hand, the effect of binding each inhibitor on the flexibility of SaSDH residues was explored through root mean square fluctuation (RMSF) plots, indicating that no important changes in the lateral chains of the amino acids were detected in this respect (Figure 4B). Finally, no effect of protein structure compactness was observed in each complex, as shown in the radius of gyration (Rg) plots (Figure 4C), where the Rg value is kept practically constant in all cases.

#### 3.5.2. Linear Interaction Energy

Finally, to understand what kind of forces dominate the binding of each inhibitor in the enzyme, the linear interaction energy was estimated from MD studies using the last 50 ns of each simulation. The data showed that in the three cases, the binding of the compounds was favorable and dominated by Van der Waals forces, with the electrostatic component playing a minor role (Table 4).

### 3.6. Physicochemical and Toxicological Properties

The parameters involved in designing bioactive molecules are the physicochemical, toxicological, and ADME properties because they play a relevant role in determining the effectiveness of a drug. These properties were determined in silico by using DataWarrior v.4.07.02 [55] and the online servers Pre-ADMET [53] and SwissADME [54]. The physicochemical properties were evaluated through Ro5, which involves the combining chemical structures with their biological activity and predicts the behavior of compounds based on the permeability of biological membranes. In this sense, a drug candidate molecule must meet specific requirements associated with molecular size, lipid solubility, polarity, electrical charges, and aqueous solubility. Ro5 considers a molecular weight greater than 500 g/mol, a number of hydrogen bond acceptors equal to or less than 10 (sum of O and N groups in the molecule), and a Log P less than 5. Therefore, it is predicted that, if a compound violates two or more conditions, it will have absorption problems.

The physicochemical parameters of the compounds were fulfilled using Lipinski’s rules. Rutin displayed a molecular weight higher than 500 g/mol, and phloridzin failed for the number of hydrogen bond donors and acceptors and rotatable bonds. Rutin also exhibited non-compliance with the CMC-like rule due to its molecular weight being higher than 480 g/mol. The CMC-like rules conform parameters such as Log P (between 0.4 and 5.6), molecular weight (160 and 480 g/mol), molar refractivity (40 and 130), and the total number of atoms (20 and 708 g/mol). On the other hand, both phloridzin and rutin were non-compliant with the lead-like rule, which established molecular weight values of 350 ± 450 g/mol and a Log P of <3, and non-compliant with the drug-like criteria, predicting that these molecules could present low absorption or permeability values. However, it was predicted that the characteristics of natural compounds or their derivatives frequently do not completely satisfy the physicochemical criteria proposed in these rules. Therefore, they are not discarded and are considered in later stages of the optimization process. Regarding the ADME properties, caffeic acid exhibited high gastrointestinal absorption. In addition, caffeic acid exhibited a high value for reproductive effects and a high risk of tumorigenicity and mutagenicity (Table 5).

## 4. Discussion

Antimicrobial resistance has become the most serious threat to human health, and infections caused by resistant bacteria are now all too common. As a result, access to effective treatments has been reduced, compelling medical professionals to use potentially more harmful, less efficient, and financially burdensome treatments. Therefore, this highlights the need to search for new options both in compounds, as well as in molecular targets.

In recent years, significant progress has been made in discovering new therapeutic strategies based on natural components derived from plant materials commonly present in the human diet [56]. Phenolic compounds are organic substances found in fruits, vegetables, cereals, roots, and leaves, and they widely described for their broad medicinal properties, such as antioxidants, anti-inflammatory agents, metabolic modulators, and antibiotics [57]. Diverse phenolic compounds with antimicrobial properties have been identified in microorganisms of clinical importance, such as *E. coli*, *K. neumoniae*, *L. monocytogenes*, *Pseudomonas aeruginosa*, and *Salmonella enterica* [58,59].

Hence, the present study aimed to explore an *in-house* natural product and synthetic derivative chemical library of *Sa*SDH inhibitors as a prospective strategy for impairing MRSA survival. Among the analyzed compounds, phloridzin exhibited the highest inhibition activity against *Sa*SDH, according to an IC_50_ value of 140 µM, while rutin and caffeic acid had IC_50_ values of 165 and 240 µM, respectively. Under molecular docking evaluation, all the compounds had affinity with the active center of the enzyme, with relevant residues found in the native catalysis of *Sa*SDH, such as Lys64 and Tyr211, increasing the effective inhibition potential, with greater affinity and specificity [60,61]. Additional interactions were observed with Leu14, Ser13, Ser15, and Gln239 residues. In most cases, hydrogen bonding interactions were identified, which may suggest that the exposed compounds could have simulated the native substrate binding mode, especially when these interactions occurred in the catalytic amino acids of *Sa*SDH [60], such as phloridzin with hydrogen bond interactions with residues of Lys64 and Tyr21. The importance of these residues within the catalysis of *Sa*SDH lies in using Lys64 as a catalytic base; similarly, Tyr211 forms hydrogen bond interactions with the natural substrate, which could provide effective competition for the active site and block catalysis [7]. On the contrary, the docking studies with the fewer active compounds in Table 3 showed that no matter which compounds performed some hydrogen bond and hydrophobic interactions, these were not made with residues important for enzyme catalysis (Table S2). This highlights the transcendence of the interactions shown by the polyphenolic compounds, which, in part, could explain their inhibitory capability. Furthermore, MD simulations showed that these compounds made stable complexes with the enzyme, at least with the conformer studied. Nevertheless, a structural analysis characterizing more than one conformer from each compound trough MD simulations would be helpful to a deeper understanding about the binding mode of these inhibitors, that is a limitation in this work. However, the structural information given here is useful as starting point for further studies with these compounds.

Moreover, a previous study demonstrated the potential of other phenolic compounds as *Sa*SDH inhibitors. Zhu and colleagues (2023) [12] evaluated two phenolic compounds ((-) Gallocatechingallate and rhodiosin) for their inhibitory capacity in the catalytic center cavity, finding hydrogen bond interactions between the ligand and Asp100, facilitating protonation [61]. Similarly, some *Sa*SDH inhibitors have been reported as having relevant hydrogen bond interactions at the binding site with the Lys64 residue [25] and cation-pi-type interactions at the Asp100 residue [26].

On the other hand, when characterizing the inhibition mechanisms of the phenolic compounds analyzed, phloridzin showed a mixed type with a predominantly competitive component, since it presented increases in *Vmax* and *Km*, indicating that phloridzin can bind to both the free enzyme and the enzyme–substrate complex. However, rutin and caffeic acid showed only a decrease in the *Vmax* value, showing that these inhibitors affect the catalysis of the enzyme by reducing the number of functional enzyme molecules that can catalyze the reaction. Therefore, it was categorized as a non-competitive mechanism, indicating the same affinity for the free enzyme as for the enzyme–substrate complex. Our research group previously described several *Sa*SDH inhibitors [25], which were shown to have a mixed partial and mixed whole inhibition mechanism, as well as an uncompetitive complete inhibition mechanism. However, the compounds presented in this study represent new chemical nuclei capable of inhibiting *Sa*SDH.

Furthermore, other natural product derivatives have been reported to have inhibitory potential on SDH in other bacterial pathogens. The effectiveness of epigallocatechin gallate and epicatechin gallate in *Pseudomonas putida* was tested, obtaining IC_50_ values of 3 and 3.7 µM, respectively [14]. On the other hand, the inhibitory potential of curcumin against SDH of *Helicobacter pylori* was described with IC_50_ values of 15.4 µM, along with a non-competitive inhibition pattern, for both shikimate and NADP^+^ [15].

Nonetheless, difficulties were encountered in determining the MIC on the reference strain, since rutin and phloridzin did not show solubility in the Mueller–Hinton medium. However, Barreca and others [62] report MIC values of DE > 1000 µg/mL within different strains of *S. aureus,* using dilution for DMSO (0.01% (*v*/*v*)). In contrast, determination could be performed using caffeic acid, which showed a 100% inhibitory effect on the growth of MRSA at a concentration of 12 mM (2.2 mg/mL). Determining MIC in potentially pathogenic bacterial strains applying phenolic compounds has been previously approved. In other studies, the effectiveness levels of compounds such as gallic, caffeic, protocatechuic, coumaric, and vanillic acids from blueberry and blackberry were tested against MRSA. The values obtained ranged from 1.3 mg/mL to 4.09 mg/mL, with caffeic acid standing out at 2.05 mg/mL. This is consistent with the results of this research, providing further support for characterizing the biological activity presented [63].

According to physicochemical and toxicological analysis, rutin has shown relevant insights. In an acute toxicity study of in vivo models, the combination of quercetin, turmeric, and rutin at doses above 2000 mg/kg/p.o. showed no significant changes in hematological parameters, lipid profiles, and biochemical parameters [64]. Additionally, caffeic acid has been previously studied, demonstrating various therapeutic applications, ranging from antioxidant to anticancer properties. However, at a specific concentration of 150 mg/kg/day, it has been shown to have the potential to generate plastic hyperplasia and possible genotoxicity [65,66]. Nevertheless, pharmacological strategies such as combining synergistic compounds can perform a dose reduction. Similarly, molecular modifications such as selective methylations can improve its stability and therapeutic potential, decreasing the pro-oxidant activity and genotoxicity [67].

Exploring therapeutic strategies based on natural product derivatives seems to be a viable option because current health problems require a more sustainable and accessible approach. Furthermore, reducing dependence on synthetic compounds can help us to develop therapies with lower toxicity, supported by new technological and biotechnology tools.

## 5. Conclusions

This study highlights three natural products that act as inhibitors of shikimate dehydrogenase. The compounds were characterized not only in vitro but also via molecular docking and molecular dynamics simulations, biological activity, and predicting their physicochemical, toxicological, and ADME properties. Taken together, these data encourage using these compounds as a starting point for developing new antibiotics based on natural products against MRSA.

## Figures and Tables

**Table 1:** The natural and synthetic product derivatives with the highest SaSDH inhibition percentages.

Compound	Structure	% Inhibition of *Sa*SDH at 500 µM
Phloridzin	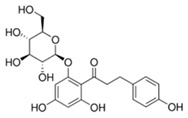	93
Rutin	*** 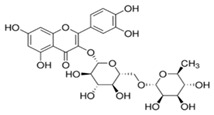 ***	87
Caffeic acid	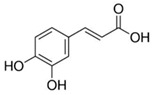	70
2,3-Diaminonaphthalene	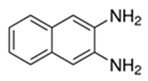	45
6-Bromo-2-hydroxy-3-methoxybenzaldehyde	*** 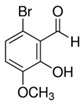 ***	45
1H-indole-2-carbaldehyde	*** 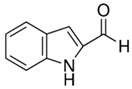 ***	34
1-(3-aminopropyl)-2-methyl-1H-imidazole	*** 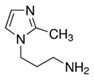 ***	34
Limonene	*** 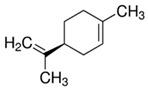 ***	32

**Table 2:** The kinetic constants and inhibition mechanisms for the three most potent inhibitors.

Compound	*Vmax* (U/mg)	*Km* (µM)	Inhibition Mechanism
*Sa*SDH Control	0.52	4	-
Phloridzin	0.25	7	Mixed with a predominant competitive component
Rutin	0.39	4	Non-competitive
Caffeic acid	0.32	4	Non-competitive

**Table 3:** The *Sa*SDH–inhibitor complex interactions formed by the three most potent compounds.

Compound	Type of Interactions				
	Hydropobic	Hydrogen Bonds	Salt Bridges	Π-Stacking	Binding Score Kcal/mol
Phloridzin	Phe236 (3.64) Asn58 (3.71) Val5 (3.89) Gln239 (3.98) Ile209 (3.99)	Tyr211 (2.85)	Phe236 (4.9)		
		Ser15 (3.0)			
		His12 (3.02)			
		Gln239 (3.04)			−8.7
		Asn58 (3.08)			
		Tyr32 (3.19)			
		Ile212 (3.26)			
		Gln239 (3.93)			
Rutin	Phe236 (3.38) Ile212 (3.48) Ile209 (3.61) Ile65 (3.64) Val5 (3.70) Ala185 (3.72) Thr60 (3.84)	Ser15 (2.79)	Lys64 (4.08)		
		Ile212 (3.06)			
		Gln239 (3.06)			
		Thr183 (3.10)			
		Asn85 (3.49)			−9.6
		His12 (3.73)			
		Lys64 (3.81)			
		Asn58 (3.99)			
		Tyr211 (4.04)			
Caffeic acid	Asn58 (3.0)	Ser15 (1.99)	Phe236 (5.38)	Lys64 (3.10)	
		Tyr32 (2.26)			
		Tyr32 (2.71)			−6.3
		Asn58 (2.82)			
		Asp100 (2.89)			
		Ser243 (3.54)			

The distance in (Å) between interacted atoms is provided within parentheses. The data were obtained using PLIP software.

**Table 4:** Binding free energies calculated using the Linear Interaction Energy Method for SaSDH–inhibitor complexes.

Complex	(V_LJ_)_bound_	(V_LJ_)_free_	(V_CL_)_bound_	(V_CL_)_free_	∆G_bind_
*Sa*SDH-Phloridzin	−40.8 ± 2.60	−13.9 ± 1.27	−8.6 ± 0.74	−12.8 ± 1.20	−2.75
*Sa*SDH-Rutin	−55.7 ± 0.36	−15.2 ± 0.31	−9.1 ± 0.45	−14.2 ± 0.36	−4.4
*Sa*SDH-Caffeic acid	−26.3 ± 0.36	−3.1 ± 0.13	−3.4 ± 0.26	−3.0 ± 0.31	−4.8

(V_LJ_)_bound_ is the average Lennard-Jones energy for protein–ligand interaction; (V_LJ_)_free_ is the average Lennard-Jones energy for ligand–water interactions; (V_CL_)_bound_ is the average electrostatic energy for ligand–protein interactions; (V_CL_)_free_ is the average electrostatic energy for ligand–water interactions; ∆G_bind_ is the binding free energy for protein–ligand interactions. Values are represented in Kcal/mol.

**Table 5:** Physicochemical and toxicological properties.

Parameters	Phloridzin	Rutin	Caffeic Acid
Physicochemical			
Molecular weight ^	436.41	610.52	180.16
Log P *	0.055	−1.25	0.78
Log S *	−2.40	−2.39	−1.40
H-bridge donors *	7	10	3
H-bridge acceptors *	10	16	4
Rotatable bonds ^	7	6	2
ADME			
Blood–brain barrier penetration ^	No	No	No
Gastrointestinal absorption ^	Low	Low	High
Plasma protein binding °	Weak	Weak	Weak
Caco2 cell permeability °	Moderate	Moderate	Moderate
Toxicological			
Irritant *	No	No	No
Effects on reproduction *	Low	No	High
Tumorigenic *	No	No	High
Mutagenic *	No	No	High
CYP450 inhibition °	2C19, 2C9 and 3A4	2C19, 2C9 and 3A4	2C9 and 3A4
Drug-Like			
Drug-likeness Score *	−4.87	1.93	0.1675
CMC-like rule °	Compliant	Non-compliant	Compliant
Lead-like rule °	Non-compliant	Non-compliant	Compliant
Ro5 °	Adequate	Non-compliant	Adequate

Log P *: Lipophilicity; lipid vs. water affinity (optimal range; −0.7 to 5.0 (SwissADME: <5)). Log S *: water solubility of the compound (>−4(ideally > −2 for good solubility)). H-bridge donors *: Atoms donating protons in hydrogen bonds (≤5 according to Ro5 criterion). H-bridge acceptors *: Atoms accepting protons in hydrogen bonds (≤10 according to Ro5 criterion). Rotatable bonds: Freely rotating single bonds (≤10 (preferred: ≤5 for oral drugs)). Blood–brain barrier penetration: Ability to cross the blood–brain barrier (Yes, for CNS drugs; No, for peripheral targets). Gastrointestinal absorption: Absorption efficiency in the digestive tract (High preferred). Plasma protein binding: Percentage bound to plasma proteins (Moderate (<90% of binding)). Caco-2 cell permeability: Permeability in the Caco-2 intestinal cell model (High preferred). Irritant *: Potential to cause tissue inflammation (No, preferred). Effects on reproduction *: Risk of harming fertility or development (No, preferred). Tumorigenic *: Potential to cause tumor formation (No, preferred). Mutagenic *: Potential to cause genetic mutations (No, preferred). CYP450 inhibition °: Inhibition of CYP enzymes; Affects drug metabolism (No strong inhibition preferred; especially CYP3A4 and CYP2D6). Druglikeness Score *. Overall suitability as a drug compound (Positive score; >0 is ideal). CMC-like rule °: Chemical modeling companion-based drug-likeness rules (compliant). Lead-like rule °: Criteria for promising early drug candidates (MW < 350; Log P < 3; HBD ≤ 3; HBA ≤ 6). Ro5 °: Lipinski’s Rule of Five for oral bioavailability (Violate ≤ 1 rule; ideal: 0 violations). (*) Determined via DataWarrior, (°) determined via PRE-ADMET, and (^) determined via SwissADME.

## Data Availability

Data are contained within the article and Supplementary Materials.

## Supplementary Materials

The following supporting information can be downloaded at https://www.mdpi.com/article/10.3390/biom15081137/s1. Figure S1. Progress kinetics inhibition curves from Phloridzin (A), Rutin (B), and Caffeic acid (C). The concentration assessed were 70 (magenta) and 100 (red) μM for Phloridzin; 100 (magenta) and 150 (red) μM for Rutin; and 100 (magenta), 150 (red), and 250 (light blue) μM for Caffeic acid. In all cases the blue line corresponds to the activity in absence of each inhibitor. Table S1. Natural and synthetic product derivatives with the SaSDH inhibition percentage. Table S2. The SaSDH-inhibitor complex interactions formed by the five less active compounds showed in Table 1.
