# Supplementary material for: Characterization of Natural Products as Inhibitors of Shikimate Dehydrogenase from Methicillin-Resistant Staphylococcus aureus: Kinetic and Molecular Dynamics Simulations, and Biological Activity Studies
Source: Biomolecules. 2025 Aug 6;15(8):1137. doi: 10.3390/biom15081137 (PMC12383815; doi:10.3390/biom15081137)
Supplement: Supplementary file 1 [file biomolecules-15-01137-s001.zip › Figure S1.pdf]

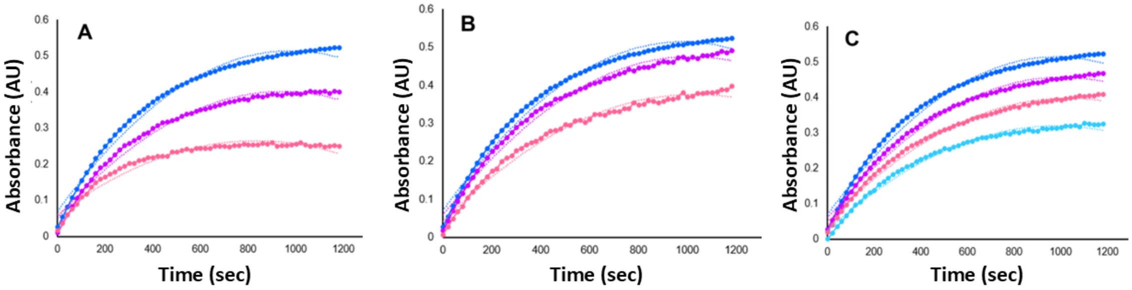

Figure S1. Progress kinetics inhibition curves from Phloridzin (A), Rutin (B), and Caffeic acid (C). The concentration assessed were 70 (magenta) and 100 (red) µM for Phloridzin; 100 (magenta) and 150 (red) µM for Rutin; and 100 (magenta), 150 (red), and 250 (light blue) µM for Caffeic acid. In all cases the blue line corresponds to the activity in absence of each inhibitor.
