# Supplementary material for: Characterization of Natural Products as Inhibitors of Shikimate Dehydrogenase from Methicillin-Resistant Staphylococcus aureus: Kinetic and Molecular Dynamics Simulations, and Biological Activity Studies
Source: Biomolecules. 2025 Aug 6;15(8):1137. doi: 10.3390/biom15081137 (PMC12383815; doi:10.3390/biom15081137)
Supplement: Supplementary file 1 [file biomolecules-15-01137-s001.zip › Table S1.pdf]

Table S1. Natural and synthetic product derivatives with the *Sa*SDH inhibition percentage.

Compound	Structure	% Inhibition of *Sa*SDH at 500 μM
Pyridoxine	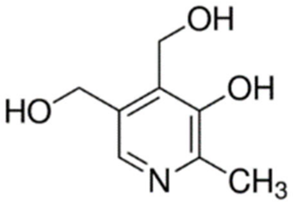	**28**
Salidroside	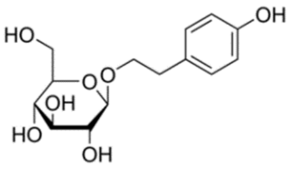	**27**
Nicotinic acid	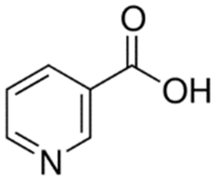	**20**
1H-Benzo[g]indole-3-carboxaldehyde	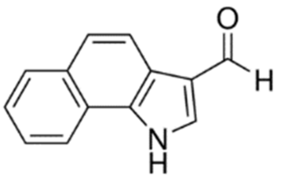	**19**
2,4-Dichloropyridine	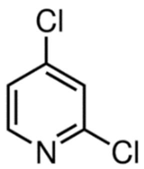	**19**

2-Chloro-5-fluoropyridine	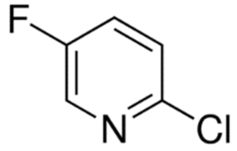	**18**
Dimethyl imidazole-4,5-dicarboxylate	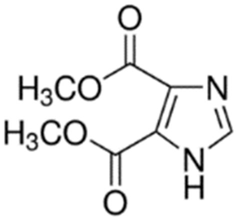	**18**
5-Bromo-2-hydroxy-3-nitropyridine	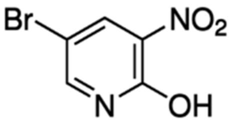	**18**
Tropane	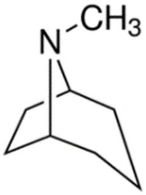	**18**
2-Aminothiazole-5-carbonitrile	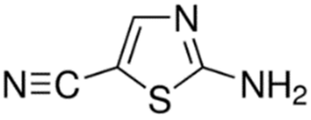	**17**
Menadione	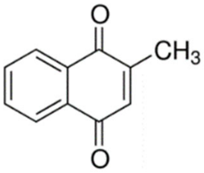	**17**
Benzothiazole-2-carbonyl chloride	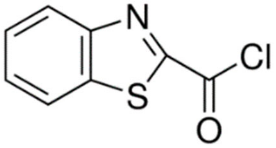	**14**

2-Oxopiperazine	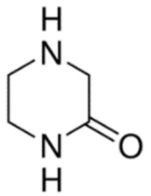	**13**
1-Methylimidazole-2-sulfonyl chloride	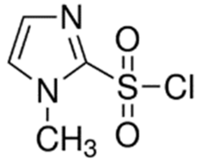	**13**
Formononetin	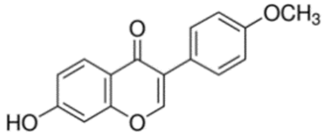	**13**
Leonurine	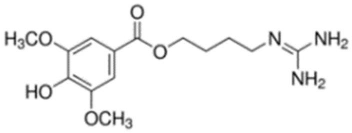	**13**
4-Deoxypyridoxine hydrochloride	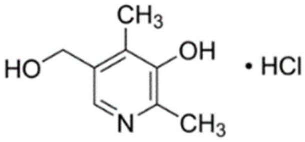	**12**
2-Bromo-1H-benzimidazole	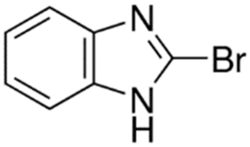	**10**
Methyl 4-methoxysalicylate	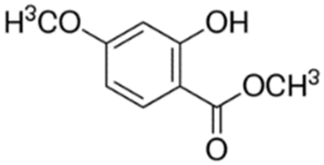	**10**

3,4-Epoxy-tetrahydrothiophene	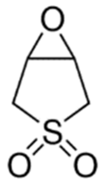	**9**
5-Bromo-2-(trifluoromethyl)pyridine	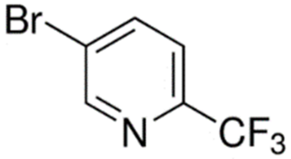	**9**
2-Amino-4-(5,6,7,8-tetrahydronaphthalen-2-yl)thiazole	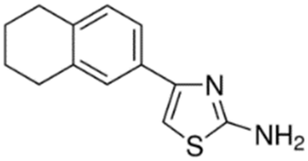	**8**
2-Amino-5-bromopyridine	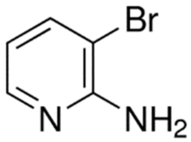	**8**
Lapachol	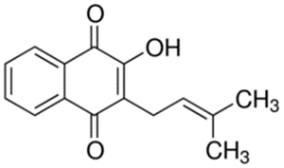	**6**
Nicotinamide	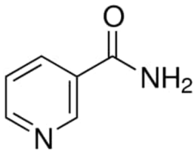	**5**
L-Ascorbic acid	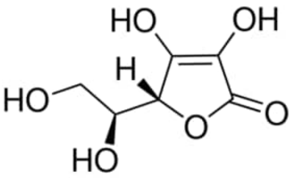	**4.5**
3-Methoxybenzoic acid	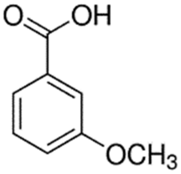	**2**

2-Hydroxy-5-(trifluoromethyl)pyridine	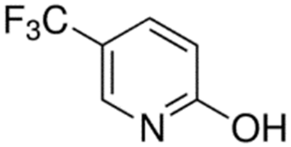	**0**
3,4-Dihydroxy-L-phenylalanine	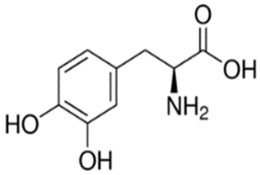	**0**
3-Bromo-5-(trifluoromethyl)pyridine	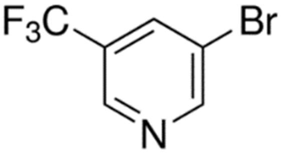	**0**
6-Chloro[1,3]dioxolo[4,5-g]quinoline-7-carboxaldehyde	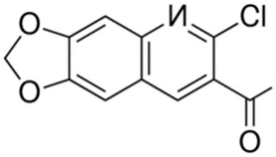	**0**
1H-Benzimidazole-2-carboxylic acid monohydrate	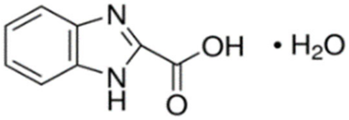	**0**
