# Supplementary material for: Characterization of Natural Products as Inhibitors of Shikimate Dehydrogenase from Methicillin-Resistant Staphylococcus aureus: Kinetic and Molecular Dynamics Simulations, and Biological Activity Studies
Source: Biomolecules. 2025 Aug 6;15(8):1137. doi: 10.3390/biom15081137 (PMC12383815; doi:10.3390/biom15081137)
Supplement: Supplementary file 1 [file biomolecules-15-01137-s001.zip › Table S2.pdf]

Table S2. The *Sa*SDH-inhibitor complex interactions formed by the five less active compounds showed in Table 1.

**Compound**	**Type of interactions**			
	**Hydropobic**	**Hydrogen bonds**	**Π-Stacking**	**Binding score Kcal/mol**
**Diaminonaphtalene**	Thr60 (3.73)<br>Asn85 (3.89)<br>Gln239 (3.95)	Asn58 (3.73)<br>Thr86 (3.02)		-6.6
**Methoxybenzaldehyde**	Thr60 (3.79)<br>Phe236 (3.48)	Lys64 (4.04)<br>Asn85 (2.84)<br>Thr99 (3.33)<br>Gln239 (3.25)		-5.7
**Carbaldehyde**	Thr60 (3.57)<br>Gln239 (3.64)	Lys64 (2.99)<br>Gln239 (3.12)	Phe236 (5.28)	-5.9
**Imidazole**	Val5 (3.49)<br>Asn58 (3.88)<br>Phe236 (3.97)	Ser15 (3.10)<br>Tyr32 (3.68)<br>Gln239 (3.13)		-5.1
**Limonene**	Thr60 (3.64)<br>Asn85 (3.79)<br>Phe236 (3.65)<br>Gln239 (3.51)			-5.4

The distance in (Å) between interacted atoms is provided within parentheses. The data were obtained using the PLIP software.
